# Enhancing trustworthiness and integrity in research

**DOI:** 10.1177/10538127251401373

**Published:** 2025-12-01

**Authors:** Remko Soer

**Affiliations:** 1 University Medical Center Groningen, Department of Anesthesiology, Pain Center, University of Groningen, Haren, The Netherlands; 2 mProve Hospitals. A strategic cooperation of seven Dutch Teaching Hospitals, Zwolle, The Netherlands

Dear colleagues,

Trustworthiness and integrity in research are becoming increasingly vital in a world defined by both exceptionally high research standards and unprecedented technological acceleration. The development of human knowledge and technology has never advanced at a faster pace, even surpassing the expectations of *Moore's Law*, which describes the exponential growth of technological capability. This rapid evolution has a profound impact on many disciplines, including pain, musculoskeletal, and rehabilitation sciences.

At the same time, our scientific community faces growing challenges: the emergence of *paper mill* research (manuscripts fabricated by commercial parties on behalf of researchers), selective reporting, lack of methodological rigor, and the persistent “publish or perish” culture. As early as 2005, Ioannidis warned us that most published research findings are, in fact, false.^1^ Nearly two decades later, his message remains as relevant as ever.

To rebuild and strengthen trust in scientific publishing, researchers are increasingly encouraged to acknowledge and correct errors, both in their own work and in that of others. Recently, editors of journals in the fields of pain and anesthesiology have endorsed the **Enhancing Trustworthiness in Pain Evidence (ENTRUST-PE)** framework.^2^ This initiative conceptualizes trustworthiness through seven foundational principles:

1. **Governance and Integrity**. Upholding principles of research integrity, complying with regulatory standards, and transparently disclosing conflicts of interest.
2. **Equity, Diversity, and Inclusivity**. Proactively integrating inclusivity strategies at the earliest stages of research design and implementation.
3. **Patient and Public Involvement and Engagement**. Embedding meaningful partnerships with individuals who have lived experience throughout the research process.
4. **Methodological Rigor**. Prioritizing high-quality, methodologically robust research with adequate power, sound analysis plans, and a focus on patient-centered outcomes.
5. **Transparency and Openness** – Adopting open research practices, including the sharing of data, materials, registration and conduct of research code.
6. **Balanced Communication** – Reporting all results accurately and comprehensively, regardless of outcome direction.
7. **Data Authenticity** – Committing to the timely correction or retraction of errors to preserve the scientific record.

The research community increasingly embraces these principles, for example through prospective study registration. While preregistration was once limited to randomized controlled trials, we now see similar efforts for other study designs including retrospective and cross-sectional studies to mitigate selective reporting bias and enhance credibility.

At the *Journal of Back and Musculoskeletal Rehabilitation (JBMR)*, we strongly align with this movement. We encourage our authors and readers to engage with the work by O’Connell and colleagues^2^ and to take note of the recent endorsement of the ENTRUST-PE framework by numerous journal editors.^3^ These efforts collectively advance the reliability and transparency of our field.

As we approach 2026, the second half of this decade, I urge all scientists to embody these principles: preregister studies, commit to methodological integrity, and work collaboratively toward a more trustworthy scientific community.

On behalf of the editorial board, I express our sincere gratitude to all contributors, authors, and readers of *JBMR*. We wish you a successful, productive, and inspiring New Year.
